# Brain–Computer Interfaces for Upper Limb Motor Recovery after Stroke: Current Status and Development Prospects (Review)

**DOI:** 10.17691/stm2023.15.6.07

**Published:** 2023-12-27

**Authors:** O.A. Mokienko, R.Kh. Lyukmanov, P.D. Bobrov, N.A. Suponeva, M.A. Piradov

**Affiliations:** MD, PhD, Researcher, Brain–Computer Interface Group of Institute for Neurorehabilitation and Restorative Technologies; Research Center of Neurology, 80 Volokolamskoe Shosse, Moscow, 125367, Russia; Senior Researcher, Mathematical Neurobiology of Learning Laboratory; Institute of Higher Nervous Activity and Neurophysiology of Russian Academy of Sciences, 5A Butlerova St., Moscow, 117485, Russia; MD, PhD, Head of the Brain–Computer Interface Group of Institute for Neurorehabilitation and Restorative Technologies; Research Center of Neurology, 80 Volokolamskoe Shosse, Moscow, 125367, Russia; PhD, Head of the Mathematical Neurobiology of Learning Laboratory; Institute of Higher Nervous Activity and Neurophysiology of Russian Academy of Sciences, 5A Butlerova St., Moscow, 117485, Russia; MD, DSc, Corresponding Member of Russian Academy of Sciences, Director of Institute for Neurorehabilitation and Restorative Technologies; Research Center of Neurology, 80 Volokolamskoe Shosse, Moscow, 125367, Russia; MD, DSc, Professor, Academician of Russian Academy of Sciences, Director; Research Center of Neurology, 80 Volokolamskoe Shosse, Moscow, 125367, Russia

**Keywords:** brain–computer interface, stroke, upper limb, neurorehabilitation

## Abstract

Brain–computer interfaces (BCIs) are a group of technologies that allow mental training with feedback for post-stroke motor recovery. Varieties of these technologies have been studied in numerous clinical trials for more than 10 years, and their construct and software are constantly being improved. Despite the positive treatment results and the availability of registered medical devices, there are currently a number of problems for the wide clinical application of BCI technologies. This review provides information on the most studied types of BCIs and its training protocols and describes the evidence base for the effectiveness of BCIs for upper limb motor recovery after stroke. The main problems of scaling this technology and ways to solve them are also described.

## Introduction

Brain–computer interface (BCI) is a technology that allows to convert data on the electrical or metabolic activity of the brain into control signals for an external technical device. In post-stroke rehabilitation, BCI is used to provide feedback to a patient during motor imagery training [1–3]. The scientific justification for this method has been the data on the positive effect of the motor imagery process on neuroplasticity due to activation of motor structures of the central nervous system (CNS) [4–8]. By providing feedback during motor imagery, the BCI systems enhance the effectiveness of such training sessions [9]. In general, training with the use of the BCI technology in patients after stroke includes the following processes: a patient is asked to mentally perform a movement of the paralyzed limb; the BCI technology using non-invasive sensors records brain signals accompanying the mental performance of the task; in real time, these signals are recognized and converted into a control command for an external device; the patient is provided with feedback on the quality of the mental task performance using the external device [10].

To date, at least 20 randomized controlled trials (RCTs) on the use of BCI for upper limb motor recovery after stroke are known worldwide, and 11 systematic reviews, 8 of which are accompanied by a meta-analysis, have been published on this topic between 2019 and 2023 [11–21]. Foreign and domestic manufacturers have developed several medical devices for use in clinical practice of post-stroke rehabilitation [22–25].

In Russia, clinical trials of BCI after stroke first began in 2011 at Research Center of Neurology (Moscow, Russia) [26, 27]. In a subsequent multicentre RCT, it was shown that a course of training with the BCI–exoskeleton complex improved the rehabilitation results of patients with focal brain damage in terms of hand motor recovery [28]. The proven technology was subsequently registered as a medical device and is currently used in a number of clinical centres [24, 29].

Despite the extensive evidence base and the availability of ready-made BCI technologies, there are currently some limitations to their widespread use in post-stroke rehabilitation, and further research and development is underway [30–37].

**The aim of this review** is to analyse scientific articles devoted to the study of the use of BCI technologies in post-stroke upper limb paresis, to outline the main problems and prospects for further development in this field.

## Literature search methodology

Articles from peer-reviewed, full-text, open access scientific journals on the use of non-invasive BCIs for upper limb motor recovery after stroke were selected for analysis. The search query was formulated according to the rules of the MEDLINE bibliographic database: ((brain–computer[tiab] OR brain–machine[tiab] OR neural interfac*[tiab]) OR “Brain–Computer interfaces”[Mesh]) AND stroke[mh] AND (upper extremity[tiab] OR hand[tiab] OR arm[tiab]). Additionally, a literature search was conducted in the eLIBRARY.RU system using the key words “brain–computer interface”, “neurocomputer interface”, “neurointerface”. The date of the search was July 3, 2023.

## Varieties of brain–computer interface systems and their application after stroke

All BCIs used in research or in the practice of post-stroke rehabilitation have distinctive features (see the Figure). The training protocols and BCI models studied in RCTs differ in the control paradigm of the interface, the type of signal recorded, the online signal processing algorithm, and the type of external technical device for providing feedback.

**Figure:**
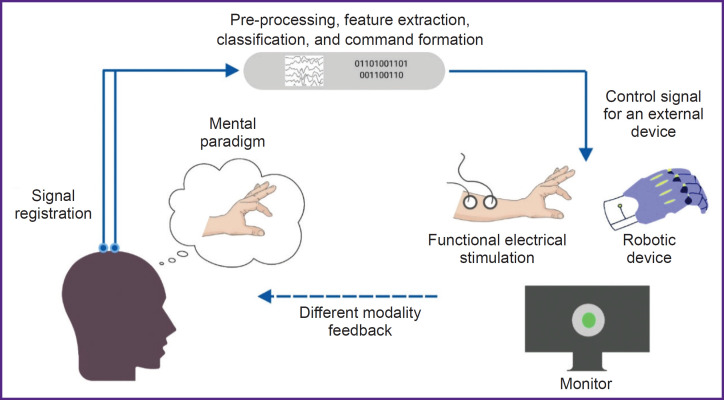
General scheme of brain–computer interface used in motor rehabilitation after stroke

### Control paradigm

Patients are typically tasked to imagine the movement, i.e., to mentally recreate the kinaesthetic sensation of a particular action in a limb without actually performing it [28, 38–45]. However, several studies have used a different paradigm — the intention to perform certain movements [46–48]. During the performance of this paradigm, in contrast to motor imagination, a patient tries to move the paralyzed limb, which is accompanied by a multiple increase in the electromyographic response compared to rest [13, 49]. In doing so, the main types of movement were clenching the hand into a fist and/or opening the hand and, less frequently, isolated or multijoint movements of the fingers, wrist, forearm, and upper arm [13].

### Control signals

Most RCTs used BCIs based on electroencephalogram (EEG) recording [11, 17], and only one used near-infrared spectroscopy (NIRS) to record brain activity signals [43]. EEG–BCIs are the most accessible varieties of this technology. As a rule, the synchronization/desynchronization response of sensorimotor rhythm over the primary somatosensory and motor cortex areas, corresponding to the process of motor imagination, is used as the recorded signal in them. In NIRS–BCIs, the sources of brain activity can be several parameters: changes in the concentration of oxy-, deoxy-, or total haemoglobin at a depth of up to 4 cm from the head surface [50]. To apply NIRS–BCIs, unlike EEG–BCIs, there is no need to use electrode gel, and the patient’s movements during training do not lead to serious signal distortions. This technology is less available than EEG–BCI and is therefore unlikely to be suitable for widespread use. However, a portable NIRS– BCI system for home use has recently been proposed and tested on a small group of patients [51]. The application of this technology at home makes it possible to extend and prolong the rehabilitation program beyond the time-limited inpatient course.

### Signal processing

Currently, there is no unified approach regarding signal processing algorithms in BCI systems. Many methods [52, 53] have been proposed and applied in various RCTs.

### External technical devices

BCI algorithms convert brain signals into control commands for external technical devices that provide real-time feedback. An orthosis, robot, or exoskeleton arm in the BCI loop performs passive limb movement that the patient represents or attempts to perform. This kinaesthetic type of feedback has been used most often in previous RCTs, including in combination with visual feedback [28, 38, 39, 41, 45, 47, 48, 54]. In a number of studies, only visual feedback in the form of an abstract signal on a computer screen was used [43, 44]. Some authors consider the functional electrical stimulation (FES) in the BCI loop to be physiologically the most preferable. During FES, more motor and sensory axons are depolarized, more powerful signals from muscles spindles and Golgi tendon organs are delivered to the CNS, and pulses from the muscle spindles can activate motor neurons simultaneously with the descending cortical command when representing a movement, thus inducing Hebbian association [13, 55–58]. The efficacy of BCI with FES has also been studied in several RCTs [42, 46, 59–61].

### Training courses

In the RCTs conducted, the frequency of BCI training sessions ranged from 2 [46] to 5 times a week [28, 45, 47, 54, 59, 60], and the total course duration ranged from 2 [28, 43] to 8 weeks [42], but most often was 4 weeks [38, 44, 45, 47, 59–61]. The total number of training sessions included from 6 [43] to 24 sessions [42], and the total training exposure ranged from 2 [43] to 27 h [39, 41].

### Patients

The population of patients with ischemic or hemorrhagic stroke in the conducted RCTs was quite heterogeneous with respect to age, disease duration, lesion localization, and degree of motor deficit. The vast majority of RCTs were conducted in Asian countries, and the authors of a recent systematic review suggest it to be inappropriate to transfer the results of these studies to older European and North American populations of post-stroke patients [11].

## Efficacy and safety of brain–computer interface technologies application after stroke

All published meta-analyses have found an advantage of BCI technologies over control groups with respect to upper limb motor function recovery after stroke as measured by Fugl-Meyer scale (see Appendix). As a rule, a medium effect size was observed, with standardized mean difference (SMD) or Hedges’ g scores greater than 0.5. The benefit of training with BCI has also been shown in terms of increased activity of daily living according to the modified Barthel index with a large effect size (SMD>1.0) [18, 20, 21].

Two studies conducted a meta-analysis in a subgroup of studies that included an additional followup period [14, 17]. An earlier study [14] found no effect in 6 weeks — 12 months after the end of the training course. In a more recent meta-analysis [17], which included a larger number of RCTs, the benefit of BCI over control groups persisted 2–36 weeks after the end of the study, but with a small effect size (SMD=0.33).

Besides, in studies evaluating recovery indices by functional magnetic resonance imaging (fMRI) or EEG, training with BCI has been shown to promote functional brain recovery with a large effect size (SMD=1.11; p<0.001) [15].

In all RCTs, no serious adverse events were reported. Some patients experienced headache, increased blood pressure, upper arm pain, skin hypersensitivity to electrode gel, and many patients experienced fatigue during training sessions. According to the meta-analysis [20], the incidence of adverse events and patient dropout rates were comparable in the BCI and control groups.

## Factors influencing the efficacy of brain–computer interface technologies

In systematic reviews [14, 21], additional subgroup meta-analyses were performed to identify possible factors influencing the efficacy of BCI (see Appendix).

Two meta-analyses evaluated the dependence of BCI efficacy on the post-stroke time. The effect size in the subgroup of patients, which had a stroke less than 6 months ago (subacute phase), was higher than in the subgroup, the patients of which had a stroke 6 or more months ago (chronic phase) [16, 20]. However, no statistically significant differences in the BCI efficacy were found between groups with different post-stroke time.

The BCI with FES, compared to BCIs connected to robotic devices or with visual feedback only, turned out to be the most effective model of this technology, as shown in four meta-analyses [14, 17, 19, 20]. All of these studies have found a large effect size (SMD or Hedges’ g >1.0) when the use of FES–BCI was compared with the control group, which used FES without BCI control.

Attempting to make a movement may be a more favourable paradigm for controlling BCI than motor imagination. Two meta-analyses have shown a trend of greater effects of BCI training using the movement-attempt paradigm [14, 17]. However, due to the statistically insignificant differences in the effect between studies with different BCI control paradigms and considering the fact that the movement-attempt paradigm was used in only two RCTs, additional studies are needed to determine the influence of this factor more precisely.

Two meta-analyses additionally studied the possible influence of selected brain signal processing algorithms on the efficacy of the BCI technology. It has been shown that the use of spectral power in a single frequency band compared to the use of filters in several bands [17], as well as the use of sensorimotor rhythm control algorithms from leads located over motor cortical areas compared to the classification of EEG from numerous leads located over the entire surface of the head, are accompanied by a larger effect size [19].

## Problems of widespread application of brain–computer interface technologies and ways to solve them

The widespread introduction of BCI technologies into clinical practice is currently hindered by a number of problems related to the technical features of existing BCI models, approaches to signal processing, and the current level of understanding the processes underlying motor recovery on the background of mental training [62–68].

One of the key challenges is the difference in individual human ability to control non-invasive BCIs using a motor imagery paradigm [69]. To master this skill, individuals usually require several BCI training sessions. However, even after training, the quality of control often remains low or instable, which demotivates patients. In addition, it has been shown that higher BCI control quality indicators are accompanied by higher motor recovery indicators [70]. At the same time, between 10 and 30% of users never achieve a proper level of BCI control. Some researchers call this phenomenon “BCI illiteracy”, while others, criticizing this term, refer to it as a “BCI inefficiency”, which can be overcome by using more efficient signal processing algorithms or sufficiently long operator training [62, 71, 72]. Most patients after stroke can control the BCI, but the quality and specificity of control depends on the degree of brain damage and neurological deficit [73, 74].

To solve the problem of the BCI control training, approaches of multiphase training of the BCI operator are being considered, where more brain signal-sensitive fMRI, transcranial electrical stimulation, or NIRS technologies are used in the first stages of motor imagery training [62, 75–77]. Developers continue to improve current signal processing approaches to increase the BCI control quality [78–84]. There is some hope for the application of deep learning algorithms in the BCI systems, including to overcome the phenomenon of “BCI illiteracy” and to ensure faster operator training [85–87]. It has also been shown that multimodal feedback (a combination of visual, auditory and somatosensory feedback) can improve the BCI control learning process [88, 89]. In cases where it is difficult for a patient to mentally imagine the movement, it is desirable to use the movement-attempt paradigm, which has been well established in some RCTs [46–48, 90].

Patient fatigue during BCI sessions is also a practical problem. Fatigue is a frequent symptom after stroke [91], and during the BCI control process it is necessary to concentrate attention for quite a long time, focusing on the mental task at hand. This problem can be overcome by providing breaks every 15 min of a training session [20], as well as by using more motivating and varied feedback in the form of a game [92, 93].

Besides, modern medical technologies should reduce the burden on health care workers and should be adapted for independent use by patients at home [94]. Most BCI developments to date do not meet these criteria. BCIs are cumbersome, require long sensor installation time and training to set up the system. Wireless high-impedance EEG systems with dry electrodes and an easy-to-operate system to launch the BCI on a mobile device can solve this problem [95–98].

With regard to the fundamental aspects of the application of rehabilitation BCIs based on motor imagery paradigm, the issue remains open as to which non-motor, non-specific mechanisms are involved in mental training-based motor recovery process. A high level of focusing on the task to control the BCI over an extended period of training may lead to an overall improvement in brain functioning, manifested by recovery of both motor and cognitive functions, which have not been adequately assessed in the majority of the RCTs conducted. Future research needs to determine whether motor learning on the background of the BCI training is a result of improvement in cognitive functions or whether the improvement in cognitive functions is secondary [62, 99, 100].

## Conclusion

From the standpoint of evidence-based medicine, training using BCI is an effective method of upper limb motor function recovery after stroke. This is particularly true for FES–BCI technologies. In addition, training using BCI involves an active motor imagery or movement-attempt paradigm, being the only active rehabilitation method for patients with severe paresis or plegia. Currently, there are a number of challenges to scaling BCI technologies in clinical practice. However, considering the shortage of personnel for classical kinesiotherapy, innovative BCI technologies remain in demand, and further developments on their basis and technical improvement are sufficiently justified.

## Appendix

**Table:** Key findings from meta-analyses of the efficacy of non-invasive brain–computer interfaces for upper limb motor recovery after stroke

References	Number of studies and patients	Key characteristics of the BCI and control groups	Key findings
Bai et al., 2020 [14]	15 RCTs and NrCTs (378 patients)	BCIs with different control paradigms (MI, MA, motor observation) and feedback types (robot, FES, visual); control — BCI simulation or standard therapy	In RCTs, the advantage of BCI over control groups (SMD=0.42; CI: 0.18–0.66; p<0.001) No statistically significant long-term effect was found for a follow-up period from 6 weeks to 12 months (SMD=0.12; CI: 0.28–0.52; p=0.54) FES–BCIs (SMD=1.04) were more efficient compared to BCIs with a robot or visual feedback (p=0.01) A trend of greater effectiveness of the MA paradigm compared to MI (p=0.07) TES does not improve the effectiveness of BCI training sessions (2 studies; SMD=0.30; CI: 0.96–0.36; p=0.37)
Kruse et al., 2020 [15]	12 RCTs (330 patients)	EEG–BCI with MI paradigm and different feedback types (robot, FES, visual); various control groups	The advantage of BCI over control groups (SMD=0.39; CI: 0.17–0.62) Training using BCI promotes functional brain recovery (larger effect size; SMD indices of recovery according to fMRI or EEG — 1.11; CI: 0.64–1.59)
Yang et al., 2021 [16]	13 RCTs (258 patients)	BCIs with different control paradigms and feedback types (unspecified); various control groups	The advantage of BCI over control groups (SMD=0.56; CI: 0.29–0.83; p<0.001) Large effect size in the subacute stroke subgroup (SMD=1.10; CI: 0.20–2.01; p=0.02) Medium effect size in the chronic stroke subgroup (SMD=0.51; CI: 0.09–0.92; p=0.02) No statistically significant differences in the BCI efficacy between the subacute and chronic stroke groups were found (p=0.24)
Mansour et al., 2022 [17]	12 RCTs (298 patients)	BCIs with MI or MA paradigm, with different feedback types; various control groups	The advantage of BCI in short-term efficacy (large effect size, Hedges’ g — 0.73) and after 2–36 weeks of the follow-up period (small effect size, Hedges’ g — 0.33) Larger effect size in studies with the MA paradigm compared to MI paradigm (Hedges’ s — 1.21 and 0.55, respectively) The largest effect size (Hedges’ g — 1.2) was found for the FES–BCI compared to BCIs connected to a robot or with visual feedback The use of power spectral density indicators in a single frequency band is accompanied by a larger effect size compared to the use of a filter bank to isolate signals in multiple frequency bands with subsequent transformation (Hedges’ g — 1.25 and –0.23, respectively)
Peng et al., 2022 [18]	16 RCTs (488 patients)	BCIs with different control paradigms (unspecified) and feedback types (unspecified); various control groups	The advantage of BCI over control groups (SMD=0.53; CI: 0.26–0.80; p<0.05) The advantage of BCI with respect to increased activity of daily living according to the Barthel index (7 RCTs; SMD=1.67; CI: 0.61–2.74; p<0.05) No advantage of BCI with respect to spasticity according to the Ashworth scale was found (6 RCTs; SMD=–0.10; CI: 0.50–0.30; p=0.61)
Nojima et al., 2022 [19]	16 RCTs and NrCTs (382 patients)	BCIs with different control paradigms and feedback types; various control groups	The advantage of BCI over control groups (SMD=0.48; CI: 0.16–0.80; p=0.006) A trend of the largest effect size was found for the FES–BCI (SMD=1.01; CI: 0.03–2.04) The use of simple control algorithms by amplitude or degree of sensorimotor rhythm suppression from leads located over motor cortex is accompanied by a larger effect size (SMD=0.74) compared to the use of more complex classification algorithms from numerous leads located over the entire surface of the head (SMD=–0.12)
Xie et al., 2022 [20]	17 RCTs (410 patients)	BCIs with different control paradigms (unspecified) and feedback types; various control groups	The advantage of BCI over control groups (SMD=0.62; CI: 0.34–0.80; p<0.0001) Large effect size in the subacute stroke subgroup (SMD=1.11; CI: 0.22–1.99; p=0.01) Medium effect size in the chronic stroke subgroup (SMD=0.68; CI: 0.32–1.03; p=0.0002) The largest effect size was found for the FES–BCI (SMD=1.11; CI: 0.67–1.54; p<0.00001) compared to BCIs connected to a robot or with visual feedback The advantage of BCI with respect to increased activity of daily living according to the Barthel index (3 RCTs; SMD=1.12; CI: 0.65–1.60; p<0.00001)
Shou et al., 2023 [21]	11 RCTs (334 patients)	BCIs with different control paradigms (unspecified) and feedback types (unspecified); in the control group — BCI simulation	The advantage of BCI over control groups according to FM–ULC (MD=4.78; CI: 1.90–7.65; p=0.001) The advantage of BCI according to the modified Barthel index (SMD=7.37; CI: 1.89–12.84; p=0.008) No advantage of BCI over control groups according to MAL, ARAT scale, and the Wolf Motor Function Test was found

H e r e: RCT — randomized controlled trial; NrCT — non-randomized controlled trial; BCI — brain–computer interface; MI — motor imagery; MA — movement attempt; FES — functional electrical stimulation; SMD — standardized mean difference; 95% CI — confidence interval; TES — transcranial electrical brain stimulation; fMRI — functional magnetic resonance imaging; EEG — electroencephalogram; FM–ULC — Fugl-Meyer Upper Limb Scale; MD — mean difference; MAL — Motor Activity Log; ARAT — Action Research Arm Test.
